# ICD-10–based screening increased detection of hospitalisations potentially eligible for paediatric palliative care consultation in the emergency department: a validation study

**DOI:** 10.1186/s12904-026-02128-7

**Published:** 2026-05-27

**Authors:** Vinícius de Mello, Marília Silveira de Almeida Campos, Natália de Araújo Neves, Thalita Zago Oliveira, João Paulo Vilela Rodrigues, Leila Costa Volpon, Marília Amaral Costa Frangiotti, Maria Olívia Barboza Zanetti, Leonardo Regis Leira Pereira, Fabiana Rossi Varallo

**Affiliations:** 1 School of Pharmaceutical Sciences of Ribeirão Preto, University of São Paulo (USP), Ribeirão Preto, 14040-900 Brazil; 2 Department of Pharmacy, State University of Maringá, Maringá, Brazil; 3 Ribeirão Preto Medical School, University of São Paulo, Ribeirão Preto, Brazil; 4 Américo Brasiliense State Hospital, Américo Brasiliense, SP Brazil

**Keywords:** Palliative Care, Emergency Service, Hospital, Paediatrics, International Classification of Diseases, Health Services Needs and Demand, Needs Assessment

## Abstract

**Background:**

Despite emergency departments (ED) having traditionally focused on disease reversal and survival goals, care in these settings also involves paediatric patients with life-limiting or life-threatening conditions (LLC/LTC) who experience acute exacerbations of their illnesses. Early identification of these patients may help address unmet needs and enhance care provision. We aimed to assess the performance of the International Classification of Diseases, 10th Revision (ICD-10)–based screening strategy to detect paediatric patients potentially eligible for paediatric palliative care (PPC) team consultation in a Brazilian ED.

**Methods:**

We conducted a cross-sectional study of 1,000 paediatric hospitalisations in the ED of a tertiary teaching hospital in Brazil (March 2020–March 2021). Potentially eligible hospitalisations were screened using Himelstein’s criteria, informed by ICD-10–based classifications of LLC/LTC, paediatric complex chronic conditions, and multidisciplinary PPC team input. Eligibility was confirmed through chart review and multidisciplinary discussion. Screening performance was evaluated using standard diagnostic accuracy measures.

**Results:**

Eighty-three hospitalisations were confirmed as potentially eligible for PPC consultation. The ICD-10–based strategy showed high sensitivity (0.99) and negative predictive value (1.00), with moderate specificity (0.73) and low positive predictive value (0.25). It increased case detection by 1.5-fold. Eligible hospitalisations were associated with younger age, higher mortality, and longer length of stay.

**Conclusions:**

ICD-10–based screening was a high-sensitivity strategy for identifying paediatric hospitalisations potentially eligible for PPC consultation. Although not sufficient as a stand-alone diagnostic approach, it may support systematic case-finding and earlier identification of unmet PPC needs, particularly in settings with limited-service integration.

**Supplementary Information:**

The online version contains supplementary material available at 10.1186/s12904-026-02128-7.

## Introduction

 Paediatric palliative care (PPC) aims to improve the quality of life of neonates, infants, children and adolescents with life-limiting or life-threatening conditions (LLC/LTC) through early identification, comprehensive symptom management, psychosocial and spiritual support for patients and families. It should be initiated at diagnosis and continue alongside disease-directed therapies, irrespective of outcome [1–3].

Despite being recognised as part of Universal Health Coverage [4], evidence shows heterogeneous PPC progress, persistent inequities, and limited access to services, particularly in low- and middle-income countries (LMIC) [5–8]. Geographic disparities, workforce constraints, and scarce data on paediatric end-of-life care have been observed in Brazil [9, 10]. PPC is largely concentrated in tertiary hospitals and is frequently delivered via inter-consultation models [9, 10], mainly for patients diagnosed with neoplasms, to achieve physical, psychological, social, and less frequently, spiritual well-being [11]. These findings not only underscore the need to prioritize education, workforce development, and research capacity [5–8] but also highlight the need to develop strategies to improve the identification [12], coverage, and timely referral of PPC within healthcare systems [13, 14].

While the emergency department (ED) is not traditionally considered an ideal setting to initiate palliative care, growing evidence shows that hospital-based physicians can effectively assist in eliciting patients’ goals of care and discussing prognosis and disease trajectory [15]. ED-initiated hospice and PPC consultations have increased significantly over time [16], suggesting an evolving role for the ED in delivering primary palliative care [17]. Research supports the early provision of palliative care in the ED to improve quality of life and reduce costs that may be associated with alternative treatments [18, 19]. It may also provide an opportunity to reduce length of stay [19, 20] and invasive interventions [20], such as cardiopulmonary resuscitation and mechanical ventilation [21].

Triggered palliative care consultations offer one way to detect patients who would benefit from palliative assistance and to connect them with services early in their care trajectory. Consensus reports recommend the use of triggers to identify patients for palliative care consultation, but no standardized criteria currently exist to guide trigger design or implementation [22]. The International Classification of Diseases, 10th Revision (ICD-10) code Z51.5 has been assessed to capture subspecialty palliative care consultations [23] among paediatric inpatients [21, 23].

Several ICD-10–based diagnostic frameworks have been developed to classify paediatric LLC/LTC in administrative or population-level data [24–27]. Their evaluations primarily addressed completeness, internal coherence, and applicability for population-level planning rather than diagnostic accuracy. None undertook formal assessment of sensitivity, specificity, or predictive values against an independent clinical reference standard, nor in emergency settings.

Thus, we aimed to assess the performance of an ICD-10–based screening strategy to detect paediatric hospitalisations with potential eligibility for PPC team consultation in a Brazilian ED.

## Methods

### Study design

A cross-sectional study was conducted to assess the performance of the ICD-10–based screening strategy developed to detect hospitalisations potentially eligible for PPC team consultation. Electronic health records (EHRs) of paediatric hospitalisations to the ED of the Hospital das Clínicas of Ribeirão Preto Medical School, University of São Paulo (HCFMRP-USP) were reviewed. The study followed the Strengthening the Reporting of Observational Studies in Epidemiology (STROBE) guidelines for cross-sectional research [28].

### Setting

The ED of HCFMRP-USP is a high-complexity teaching hospital and a regional reference centre for emergency care in cases of paediatric patients presenting with acute clinical conditions, stroke, exogenous intoxications, venomous animal accidents, and trauma. It comprises 51 support beds and 137 active beds, including 25 allocated to paediatric care: 14 in the Paediatrics Ward, five in the Infectious Diseases Ward, and eight in the Paediatric Intensive Care Unit. The PPC team, formally established in March 2020, includes a physician trained in PPC, a pharmacist, a nurse, a psychologist, a physiotherapist, and an occupational therapist, with weekly multidisciplinary meetings. PPC operates as a consultation service, initiated by assistant physicians based on clinical judgment, and conducted by a paediatrician trained in PPC. Each case requiring PPC consultation is reviewed in multidisciplinary meetings, tailored to specific needs and the availability of paediatric professionals. Participants may include ward and intensive care paediatricians, surgeons, pharmacists, nurses, psychologists, physiotherapists, occupational therapists, dietitians, speech therapists, social workers, and chaplains.

### Study size

The sample size was calculated based on the formula proposed by Medronho et al. [29], with an absolute error of 0.05, a significance level of 5%, and a finite population correction. The predictor variable for the calculation was the estimated prevalence of paediatric hospitalisations with at least one complex health condition (55.0%) [21]. Based on these parameters, the minimum required sample size was 809 hospitalisations.

### Participant recruitment

A total of 1,000 paediatric hospitalisations were randomly selected from all ED cases recorded between 1 March 2020 and 31 March 2021. Eligibility required age < 18 years and a length of stay of at least 24 h. Readmissions occurring within the study period were considered independent hospitalisation events.

Potential eligibility for PPC team consultation was assessed using a systematic ICD-10–based screening strategy. The screening framework incorporated the general care codes Z51 (other medical care) and Z51.5 (palliative care), together with a predefined list of diagnostic codes associated with LLC/LTC. The selection of diagnostic was based on the criteria proposed by Himelstein et al. [2] and further refined using previously published ICD-10–based classifications of paediatric LLC/LTC, including the Directory developed by Hain et al. [26], epidemiological approaches using administrative data [25], and classifications of paediatric complex chronic conditions [24]. The final list was iteratively developed and adapted with input from the multidisciplinary PPC team to reflect clinically relevant conditions in the ED setting (Appendix 1).

ICD-10 codes recorded at admission, during hospitalisation, and at discharge were considered in the screening strategy. In our setting, ICD-10 codes are recorded within the EHR and assigned during clinical documentation throughout the hospitalisation, becoming progressively available as the patient’s condition evolves.

Following the screening process, hospitalisations were allocated into two analytical categories. The first comprised cases with evidence of potential eligibility for PPC team consultation, defined by the presence of at least one relevant ICD-10 code and/or a formal PPC consultation. The second comprised hospitalisations without evidence of potential eligibility, characterized by the absence of trigger ICD-10 codes and the absence of consultation records. For both cohorts, the research team conducted detailed chart reviews and multidisciplinary clinical discussions to validate the ICD-10–based screening strategy.

The following definitions were adopted in this study: LLC are those for which there is no reasonable hope of cure and from which children will die. Some conditions cause progressive deterioration, meaning that the child becomes increasingly dependent on parents and carers. LTC are those for which curative treatment may be feasible but may fail. Palliative care for children and young people with LLC/LTC is an active and total approach to care, from the point of diagnosis or recognition throughout the child’s life and death [30].

### Variables, data sources, measurement, and data collection

**Fig. 1:**
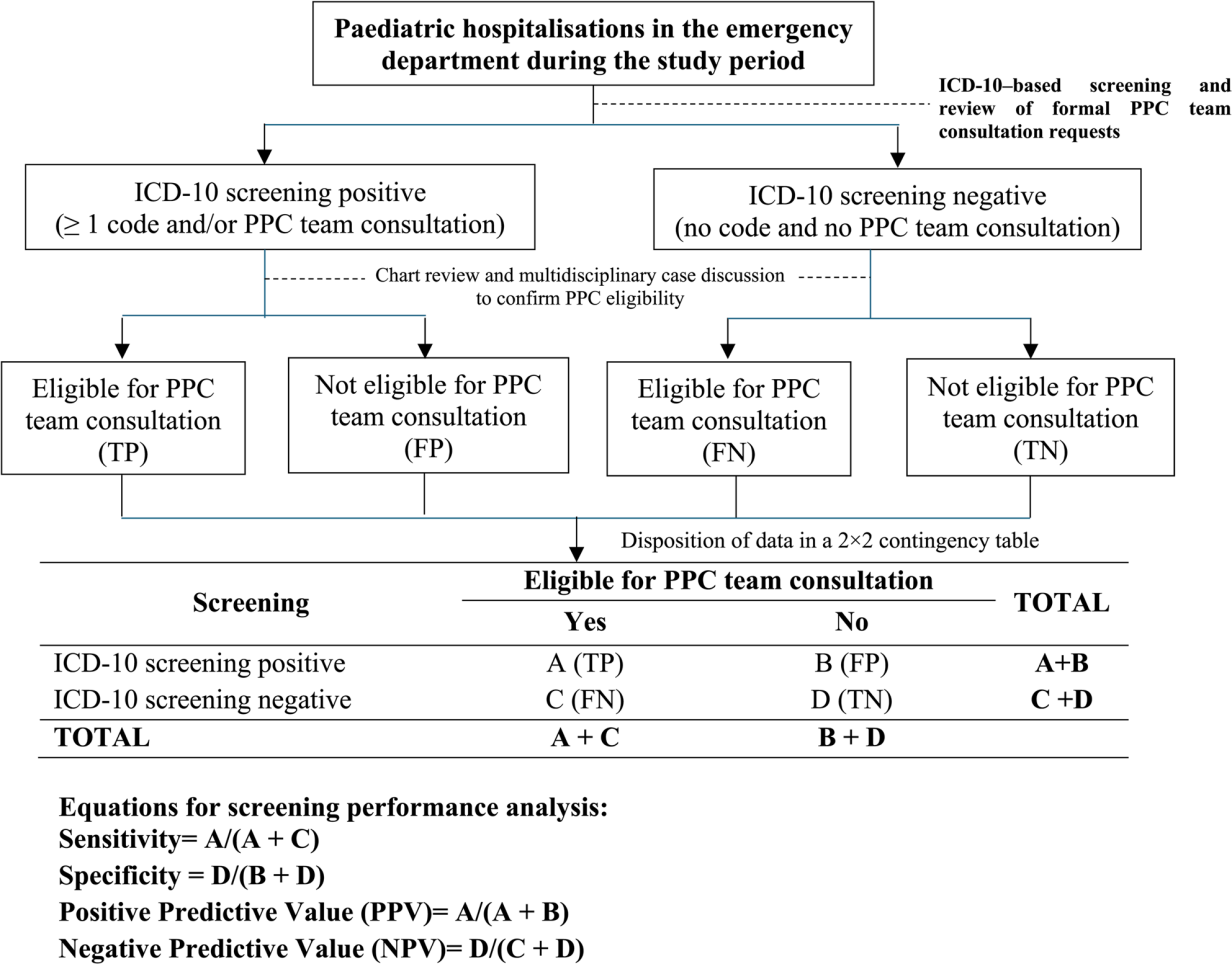
Flowchart of the ICD-10–based screening strategy and analytical framework used to evaluate its performance in identifying paediatric hospitalisations potentially eligible for PPC team consultation in the emergency department

The primary variable of interest was the validation of ICD-10 codes in screening hospitalisations potentially eligible for PPC team consultation. Secondary variables included demographic characteristics (sex and age) and clinical parameters (diagnostic group, length of hospital stay, and mortality). All data were extracted from the institutional EHR. For performance analyses (validation), the following definitions were applied:

- False positives (FP): hospitalisations identified with at least one relevant ICD-10 code in which the patient was not eligible for PPC.
- False negatives (FN): hospitalisations without any relevant ICD-10 code in which the patient was potentially eligible for PPC team consultation [21].
- True positives (TP): hospitalisations screened positive by the ICD-10 strategy and confirmed as potentially eligible for PPC team consultation after chart review and multidisciplinary discussion.
- True negatives (TN): hospitalisations without any ICD-10 codes of interest and/or no formal PPC team consultation, in which the patient was not eligible for PPC (Figure 1).

Eligibility determinations were based on the information documented in the EHR. In cases of incomplete or ambiguous records, the PPC team used clinical judgment to determine potential eligibility.

Diagnostic test characteristics [31] [sensitivity, specificity, positive predictive value (PPV), negative predictive value (NPV)], and likelihood ratios, were calculated from TP, FP, FN and TN classifications and were reported with 95% confidence intervals (CIs). Validation analyses were also performed by age group (neonates: 0–30 days; infants: 31 days to < 2 years; children: 2–12 years; adolescents: 13–18 years).

### Statistical analysis

Descriptive statistics were used for demographic variables. Frequencies were calculated for categorical variables, while means and standard deviations (SD) were computed for continuous variables. In addition, age and length of hospital stay were treated as continuous variables and summarized as median and interquartile range. As both variables were non-normally distributed, comparisons between patients who were eligible for palliative care and those who did not were performed using the Mann–Whitney U test. Associations between potential eligibility for PPC team consultation and the binary variables sex and mortality were evaluated using odds ratios (ORs) with 95% confidence intervals (95% CIs), and p-values were obtained using Fisher’s exact test. A significance level of 5% was adopted for all analyses, which were performed in Python version 3.13.9 using the Spyder integrated development environment (IDE).

### Ethics approval

The requirement for informed consent was waived by the Institutional Review Board named Research Ethics Committee of the School of Pharmaceutical Sciences of Ribeirão Preto, University of São Paulo. The waiver of informed consent was granted on the basis that the study used anonymized, retrospective data, posing no foreseeable risk to participants and complying with all applicable regulatory and institutional standards.

The study was conducted in full accordance with the principles of the Declaration of Helsinki. Ethical approval was obtained from the Research Ethics Committee of the School of Pharmaceutical Sciences of Ribeirão Preto, University of São Paulo (CAAE nº 40048720.3.0000.5403, process nº 4.527.068), and from the Research Ethics Committee of the Hospital das Clínicas, Ribeirão Preto Medical School (HCFMRP-USP) (CAEE nº 40048720.3.3001.5440, process n° 4.531.528.

## Results

During the study period, 2,260 paediatric hospitalisations occurred, of which 1,000 were randomly selected for analysis. Of these, 33.0% (330/1,000) contained at least one ICD-10 code included in the screening framework. Following chart review and multidisciplinary assessment, 82 of these cases were confirmed as potentially eligible for PPC team consultation.

**Fig. 2:**
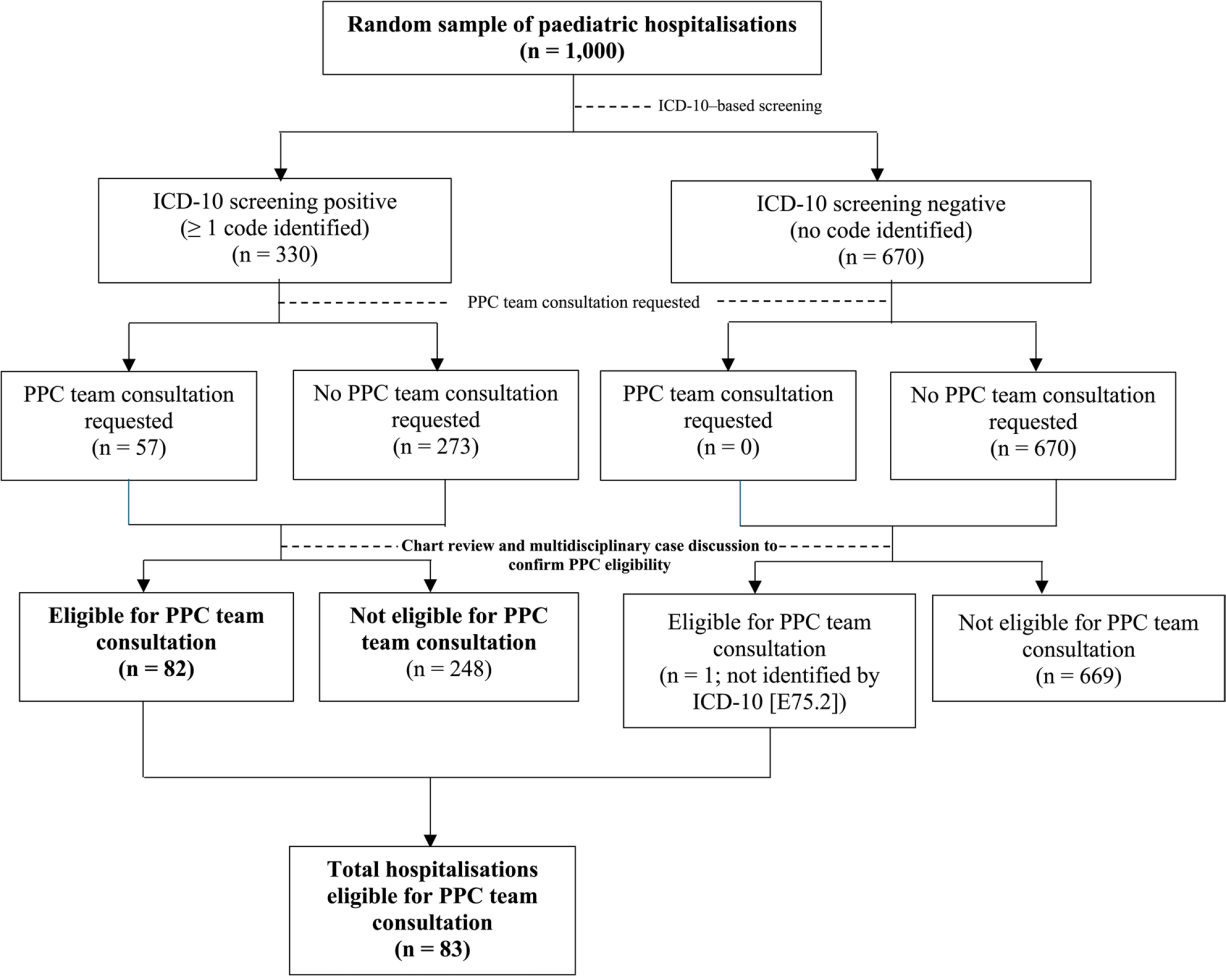
Screening and eligibility flowchart of the ICD-10–based strategy for detecting paediatric hospitalisations potentially eligible for PPC team consultation in the Emergency Department of HCFMRP-USP. Legend: ICD-10: International Classification of Diseases, 10th Revision; PPC: Paediatric Palliative Care

One additional hospitalisation was not identified by the screening strategy. This case included the ICD-10 code E75.2 (other sphingolipidosis) and was subsequently classified as eligible after chart review and multidisciplinary discussion. Overall, 8.3% (83/1,000) of hospitalisations were confirmed as potentially eligible for PPC team consultation (Fig. 2).

Overall, 31.3% (26/83) of hospitalisations with confirmed potential eligibility had no record of PPC team consultation (Fig. 2). Therefore, in addition to the 57 hospitalisations already attended by the PPC team, the expanded ICD-10 trigger list would have identified 26 additional potentially eligible cases, representing an approximately 1.5-fold increase in detection after clinical validation (Fig. 2).

Among the 82 hospitalisations identified by ICD-10 codes and confirmed as potentially eligible for PPC team consultation, 51 distinct ICD-10 codes were recorded, totalling 177 occurrences (Table 1). The most frequent codes were related to central nervous system conditions (*n* = 91), congenital malformations, deformations, and chromosomal abnormalities (*n* = 48), and endocrine, nutritional and metabolic diseases (*n* = 14) (Table 1).

**Table 1:** Frequency of ICD-10 codes detected in paediatric hospitalisations confirmed as eligible for paediatric palliative care team consultation in the Emergency Department, 2020–2021 (*n* = 177)

CODE	SHORT DESCRIPTION	Frequency (*n*)
Neoplasms		
C48.0	Malignant neoplasm of retroperitoneum	1
C49.4	Malignant neoplasm of connective and soft tissue of abdomen	1
C71.0	Malignant neoplasm of cerebrum, except lobes and ventricles	1
C74.9	Malignant neoplasm of unspecified adrenal gland	1
D43.2	Neoplasm of uncertain behaviour of brain, unspecified	1
Subtotal		5
Diseases of the blood and blood-forming organs and certain disorders involving the immune mechanism		
D57.1	Sickle-cell disease with acute chest syndrome	2
D84.9	Immunodeficiency, unspecified	1
Subtotal		3
Endocrine, nutritional and metabolic diseases		
E23.2	Diabetes insipidus	6
E71.3	Disorder of fatty-acid metabolism, unspecified	3
E72.5	Disorders of glycine metabolism	1
E83.5	Disorders of calcium metabolism	2
E88.8	Other specified metabolic disorders	1
E88.9	Metabolic disorder, unspecified	1
Subtotal		14
Diseases of the nervous system		
G04.9	Encephalitis, unspecified	1
G37.9	Demyelinating disease of central nervous system, unspecified	1
G40.1	Focal epilepsy and epileptic syndromes with localised onset seizures	16
G40.4	Other generalised epilepsy and epileptic syndromes	7
G40.9	Epilepsy, unspecified	23
G41.0	Status epilepticus, generalised tonic-clonic	3
G41.9	Status epilepticus, unspecified	3
G71.3	Mitochondrial myopathy, not elsewhere classified	2
G80.0	Spastic quadriplegic cerebral palsy	5
G80.2	Spastic hemiplegic cerebral palsy	1
G80.3	Athetoid cerebral palsy	2
G80.8	Other cerebral palsy	10
G82.4	Spastic tetraplegia	1
G91.0	Communicating hydrocephalus	2
G91.9	Hydrocephalus, unspecified	10
G93.4	Encephalopathy, unspecified	4
G94.1	Hydrocephalus in neoplastic disease	1
Subtotal		91
Diseases of the circulatory system		
I42.8	Other cardiomyopathies	1
Subtotal		1
Diseases of the genitourinary system		
N18.9	Chronic kidney disease, unspecified	1
Subtotal		1
Certain conditions originating in the perinatal period		
P21.0	Severe birth asphyxia	1
Subtotal		1
Congenital malformations, deformations and chromosomal abnormalities		
Q02	Microcephaly	8
Q03.1	Atresia of foramina of Magendie and Luschka	5
Q03.9	Congenital hydrocephalus, unspecified	9
Q04.0	Congenital malformations of corpus callosum	1
Q04.2	Holoprosencephaly	4
Q04.3	Other reduction deformities of brain	1
Q04.8	Other specified congenital malformations of the brain	2
Q04.9	Congenital malformation of brain, unspecified	1
Q05.2	Lumbar spina bifida with hydrocephalus	2
Q05.9	Spina bifida, unspecified	1
Q07.0	Arnold-Chiari syndrome	5
Q24.8	Other specified congenital malformations of heart	1
Q75.3	Macrocephaly	1
Q78	Other osteochondrodysplasias	1
Q87.8	Other specified congenital malformation syndromes not elsewhere classified	2
Q89.9	Congenital malformation, unspecified	3
Q96.9	Turner syndrome, unspecified	1
Subtotal		48
Factors Influencing Health Status and Contact with Health Services		
Z51.5	Palliative care	12
Subtotal		12
Total		177

ICD-10 codes and descriptions were based on the World Health Organization (WHO) ICD-10 classification

The high frequency of codes reflects the presence of multiple relevant diagnoses within individual hospitalisation episodes. For example, ICD-10 code N18.9 was recorded alongside Q05.9, while E23.2 co-occurred with G40.9 or C48.0.

Z51.5 ICD-10 code was recorded in 16.0% of hospitalisations confirmed as potentially eligible for PPC team consultation (12/82), all of which had a documented PPC team consultation.

The overall screening strategy demonstrated high sensitivity (0.99) and negative predictive value (1.00), with moderate specificity (0.73) and low positive predictive value (0.25). Upon subgroup analysis, the strategy showed superior performance in infants (Table 2).

**Table 2:** Performance of an ICD-10–based screening strategy to detect paediatric hospitalisations potentially eligible for paediatric palliative care team consultation in the Emergency Department. Ribeirão Preto, São Paulo, 2020–2021 (*n* = 1,000)

Subgroup	Hospitalisations (*n*)	Triggered Hospitalisations *n*	TP	FP	TN	FN	Sensitivity (CI 95%)	Specificity (CI 95%)	PPV (CI 95%)	NPV (CI 95%)
Neonates	5	0	0	0	5	0	NC	1.00 (0.48;1.00)	NC	1.00 (0.48;1.00)
Infants	198	82	27	55	116	0	1.00 (0.87;1.00)	0.68 (0.60;0.75)	0.33 (0.23;0.44)	1.00 (0.97;1.00)
Children	621	193	49	144	427	1	0.98 (0.89;1.00)	0.75 (0.71;0.78)	0.25 (0.19;0.32)	1.00 (0.99; 1.00)
Adolescents	176	55	6	49	121	0	1.00 (0.54; 1.00)	0.71 (0.64; 0.78)	0.11 (0.04; 0.22)	1.00 (0.97; 1.00)
Overall	1000	330	82	248	669	1	0.99 (0.93; 1.00)	0.73 (0.70; 0.76)	0.25 (0.20; 0.30)	1.00 (0.99; 1.00)

Legend: *CI* Confidence interval, *FN* false negative, *FP* false positive, *ICD-10* International Classification of Diseases 10th, *NC* not calculated, *NPV* Negative Predictive Value, *PPV* Positive predictive Value, *TN *True Negative, *TP* True Positive

Most patients eligible for PPC consultation were children [60.2% (50/83)], with a mean age of 62.7 months (SD ± 52.8; approximately 5.2 years), and an average length of hospital stay of 22.0 days (SD ± 22.9) (Table 3). No significant differences in sex distribution were identified. A higher proportion of deaths was observed among hospitalisations with confirmed potential eligibility for PPC team consultation. These hospitalisations also involved younger patients and were associated with longer hospital stays (Table 3).

**Table 3:** Risk factors associated with paediatric hospitalisations confirmed as potentially eligible for paediatric palliative care team consultation in the Emergency Department (*n* = 1,000)

Characteristic	Eligible for PPC		OR (CI 95%)	Median difference (CI 95%)	*p*-value
	Yes 83 (8.3%)	No 917 (91.7%)			
Sex					1.000
Male	46	512	1.01 (0.65; 1.58)	N/A	
Female	37	405			
Mortality					< 0.001**
No	66	911	39.11 (14.92; 102.52)	N/A	
Yes	17	6			
Age (months)					< 0.001*
Median (Q1-Q3)	48 (12–95)	77 (28–139.25)	N/A	-29.0 (-42.5; -7.0)	
Length of hospital stay (days)					
Median (Q1-Q3)	11 (4–33.25)	3 (2–5)	N/A	8 (5; 16)	< 0.001*

Legend: *CI* Confidence Interval, *N/A *Not Applicable, *OR* Odds Ratios, *PPC* Paediatric Palliative Care, *Q1* first quartile, *Q3* third quartile. *Significance in the Mann–Whitney U test. **Significance in the Fisher’s exact test

## Discussion

The ICD-10–based screening strategy demonstrated a performance profile aligned with PPC system requirements. It showed high sensitivity and very high negative predictive value, identifying nearly all hospitalisations potentially eligible for PPC team consultation. This was achieved despite moderate specificity and low positive predictive value. As sensitivity and specificity are independent of disease prevalence, whereas predictive values are not [32], the low prevalence of LLC/LTC and the heterogeneity of the ED population likely explain these findings.

This profile is consistent with the expected behaviour of screening strategies, which prioritise sensitivity to minimise false negatives, even at the expense of identifying additional cases requiring further evaluation [33, 34]. In PPC, under-recognition and delayed referral remain persistent challenges, particularly in low- and middle-income countries with limited access to services [8, 35–37]. In this context, prioritising sensitive case-finding approaches may support earlier identification of children potentially eligible for PPC team consultation.

Our ICD-10 list aligns with four internationally used frameworks for classifying paediatric LLC/LTC [24–27]. However, these frameworks were developed primarily for population-level epidemiological analyses rather than real-time clinical screening or individual-level case finding [38].

Similarly, administrative data–based approaches in adult populations, including ICD-10–based methods [39] and mortality prediction models such as the Hospital One-Year Mortality Risk (HOMR) [40], have been used for population-level estimation. Modified versions (mHOMR), applied in hospitalised patients, have shown a high prevalence of unmet palliative care needs and have been explored as triggers for referral in clinical settings [41]. However, these models rely on routinely collected data and require adaptation for clinical use, as their original design does not support real-time decision-making at admission.

Accordingly, our strategy extends these approaches to a clinical screening context. It evaluates ICD-10 codes across emergency hospitalisations, validates performance against multidisciplinary clinical review, and positions ICD-based screening as a complementary prescreening step rather than a substitute for bedside clinical assessment.

Eligibility instruments constitute a central component of early identification strategies in PPC [42]. These tools support the identification of patients who may benefit from PPC and guide decisions regarding the level of care provision, ranging from a palliative approach to generalist or specialized PPC [42]. Their application is not limited to the initial assessment. Repeated use throughout the disease trajectory allows clinicians to detect changes in clinical status or care complexity that may warrant reassessment or referral to a different level of PPC [42].

Among the eligibility instruments most consistently cited in international literature, the Paediatric Palliative Screening Scale (PaPaS) [43, 44] and the Assessment Form for Complex Clinical Needs in Paediatrics (ACCAPED) [45, 46] represent structured approaches for early identification and referral [42]. The PaPaS scale quantifies illness burden affecting the child and family. The ACCAPED scale evaluates clinical and care complexity across multiple domains. Both tools generate composite scores that support stratification and guide referral to the level of PPC most appropriate to the child’s needs [42]. The “surprise question” (“Would I be surprised if this child died within the next 12 months?”) may serve as an adjunctive prognostic prompt, although it does not replace systematic assessment [47–49].

Compared with bedside instruments, ICD-10 diagnostic coding offers distinct advantages in epidemiological frameworks. ICD-10 classifications are routinely collected in health systems and have been used internationally to support standardized identification and population-level surveillance of paediatric LLC/LTC [24–27, 38]. Although not originally designed for real-time clinical screening, their universal availability within electronic health records allows ICD-10–based approaches to function as an initial, broad case-finding strategy, complementing bedside tools that require multidimensional clinical assessment by specialized PPC teams [2, 42].

The selection of diagnostic codes is also critical. Some paediatric studies rely on ICD-10 code Z51.5 as a proxy for PPC consultation [21, 23]. However, this code is infrequently used to identify patients potentially eligible for PPC [50]. Among paediatric hospitalisations, only 4% of critically ill children who were potentially eligible for PPC received any ICD-10 code indicating specialist consultation (Z51.5) [21]. Evidence from Brazilian physicians supports this finding, indicating that discomfort with end-of-life discussions, limited PPC training, uncertainty regarding referral criteria, and restricted availability of specialized services reduce the likelihood of PPC documentation, thereby contributing to the low use of Z51.5 in practice [51].

Despite these limitations, O’Keefe and Czaja [21] reported good specificity but limited sensitivity of administrative codes when compared with PPC consultation registries. Goldberg et al. [23] demonstrated high accuracy of Z51.5 among paediatric decedents. Although useful for identifying hospitalisations that received PPC consultation, Z51.5 captures only cases already referred to specialist teams or those who died. Exclusive reliance on this code risks failing to detect hospitalisations potentially eligible for PPC team consultation. Our approach contributes to identifying PPC-relevant hospitalisations regardless of survival or documented referral, thereby reducing missed opportunities for earlier integration of PPC [38].

The clinical profile of hospitalisations confirmed as potentially eligible for PPC team consultation supports the relevance of this strategy in the emergency setting. These hospitalisations involved younger patients, longer lengths of stay, and a higher proportion of deaths, suggesting that the ICD-10–based approach was able to identify a subgroup with greater clinical complexity and vulnerability. The 1.5-fold increase in case detection also deserves attention, as it indicates that reliance on usual referral practices alone may leave a considerable proportion of potentially eligible children unrecognised.

Within this context, our ICD-10–based strategy may function as an automated pre-screening tool rather than a stand-alone method for determining eligibility. Because it relies on routinely collected diagnostic data, it can be integrated into hospital information systems to continuously identify hospitalisations with potential PPC needs without increasing clinician workload.

This study also highlights the role of neurological conditions, particularly epilepsy, as frequent triggers of potential PPC eligibility [25, 26]. Epilepsy, especially in refractory or syndromic forms, is associated with high symptom burden, neurodevelopmental impairment, and frequent hospitalisations. These characteristics align with PPC principles and reinforce the importance of including neurological ICD-10 codes in screening strategies [52–54]. The mean age of hospitalisations confirmed as potentially eligible for PPC team consultation was lower than that reported in other paediatric ED cohorts [55, 56]. Differences across studies may reflect variations in paediatric age definitions across healthcare systems.

A higher proportion of deaths and longer lengths of stay were observed among hospitalisations confirmed as potentially eligible for PPC team consultation. According to the PPC protocol in the ED (UE-HCFMRP-USP, 2021), consultation initially focused on patients with the greatest clinical complexity, which may explain these findings. Prolonged hospital stays may also reflect the challenges of ensuring safe discharge for complex patients in resource-limited settings. PPC care planning requires not only clinical stability but also coordinated attention to the social, spiritual, and ethical needs of patients and families to support an appropriate transition of care. The multidisciplinary team plays a central role in ensuring that all requirements for safe discharge are met [57].

### Limitations

This study has limitations inherent to ICD-based approaches. ICD-10 codes suggestive of LLC/LTC are not exhaustive and require periodic updating. The strategy depends on coding accuracy and completeness, was developed and validated in a single tertiary ED, and relies primarily on diagnostic information rather than dynamic indicators of clinical complexity. PPC implementation at the study institution is relatively recent, which may have influenced data standardization. Documentation inconsistencies and the COVID-19 pandemic may also have affected data quality and care processes.

Despite these limitations, this strategy advances the use of ICD-based methodologies from population-level estimation to clinical screening in a middle-income setting. This was achieved by validating ICD-10 codes against multidisciplinary chart review and by applying them beyond mortality-based approaches. When integrated into hospital information systems and combined with clinical instruments, this approach may support a two-step model. This would involve ICD-10 pre-screening followed by focused clinical assessment and PPC team consultation or goal-of-care planning. In turn, this may contribute to more timely identification and improved access to care for paediatric patients with potential LLC/LTC.

## Conclusion

This ICD-10–based screening strategy effectively identified paediatric hospitalisations with potential LLC/LTC and eligibility for PPC team consultation, including living patients with unmet needs who would not be captured using mortality-based approaches alone. Neurological and epilepsy-related conditions were prominent among identified cases. Although not a substitute for multidimensional clinical assessment, ICD-10–based screening represents a practical and scalable pre-screening strategy, particularly in health systems where PPC integration remains limited.

## Supplementary Information

Supplementary Material 1

## Data Availability

The dataset used and/or analysed during the current study are available from the corresponding author on reasonable request.
