# Supplementary material for: ICD-10–based screening increased detection of hospitalisations potentially eligible for paediatric palliative care consultation in the emergency department: a validation study
Source: BMC Palliat Care. 2026 May 27;25:216. doi: 10.1186/s12904-026-02128-7 (PMC13393898; doi:10.1186/s12904-026-02128-7)
Supplement: Supplementary file 1 — Supplementary Material 1 [file 12904_2026_2128_MOESM1_ESM.docx]

**Table 1**. Selected ICD-10 codes, based on Himelstein et al. (2004) criteria, for screening potential life-limiting conditions eligible for pediatric palliative care consultation.

| **Category** | **ICD-10 Code** | **Description** |
| --- | --- | --- |
| Palliative Care | Z51.5 | Palliative care |
| Chronic Respiratory Failure | J96.1 | Chronic respiratory failure |
| Severe Cerebral Palsy | G80.2 | Spastic hemiplegic cerebral palsy |
| Severe Cerebral Palsy | G82.4 | Spastic tetraplegia |
| Severe Cerebral Palsy | G80.8 | Other infantile cerebral palsy |
| Severe Cerebral Palsy | G80.3 | Dyskinetic cerebral palsy |
| Severe Cerebral Palsy | G80.0 | Spastic quadriplegic cerebral palsy |
| Severe Cerebral Palsy | G80.1 | Spastic diplegic cerebral palsy |
| Neuromuscular Diseases | G71.3 | Mitochondrial myopathy, not elsewhere classified |
| Epilepsy / Status Epilepticus | G41.9 | Status epilepticus, unspecified |
| Epilepsy / Status Epilepticus | G40.4 | Other generalized epilepsies |
| Epilepsy / Status Epilepticus | G41.0 | Grand mal status epilepticus |
| Epilepsy / Status Epilepticus | G40.9 | Epilepsy, unspecified |
| Epilepsy / Status Epilepticus | G40.1 | Localization-related symptomatic epilepsies |
| Severe Anoxia | G41.9 | Status epilepticus, unspecified |
| Severe Anoxia | P21.0 | Severe birth asphyxia |
| Severe Anoxia | G40.4 | Other generalized epilepsies |
| Severe Anoxia | G41.0 | Grand mal status epilepticus |
| Severe Anoxia | G40.9 | Epilepsy, unspecified |
| Severe Anoxia | G40.1 | Localization-related symptomatic epilepsies |
| Severe Anoxia | G93.1 | Anoxic brain damage |
| Severe Anoxia | P21.9 | Birth asphyxia, unspecified |
| Brain & Spinal Malformations | G91.1 | Obstructive hydrocephalus |
| Brain & Spinal Malformations | Q03 | Congenital hydrocephalus |
| Brain & Spinal Malformations | Q03.9 | Congenital hydrocephalus, unspecified |
| Brain & Spinal Malformations | Q04.9 | Congenital malformation of brain, unspecified |
| Brain & Spinal Malformations | Q03.1 | Atresia of foramina of Luschka and Magendie |
| Brain & Spinal Malformations | Q87.8 | Other congenital malformation syndromes |
| Brain & Spinal Malformations | G91 | Hydrocephalus |
| Brain & Spinal Malformations | Q04.2 | Holoprosencephaly |
| Brain & Spinal Malformations | Q04.8 | Other congenital malformations of brain |
| Brain & Spinal Malformations | Q75.3 | Macrocephaly |
| Brain & Spinal Malformations | Q02 | Microcephaly |
| Brain & Spinal Malformations | Q07.0 | Arnold–Chiari syndrome |
| Brain & Spinal Malformations | G91.9 | Hydrocephalus, unspecified |
| Brain & Spinal Malformations | Q89.9 | Congenital malformation, unspecified |
| Brain & Spinal Malformations | Q05.2 | Lumbar spina bifida with hydrocephalus |
| Brain & Spinal Malformations | G91.0 | Communicating hydrocephalus |
| Brain & Spinal Malformations | G91.8 | Other hydrocephalus |
| Brain & Spinal Malformations | G94.1 | Hydrocephalus in neoplastic disease |
| Brain & Spinal Malformations | Q03.0 | Malformations of aqueduct of Sylvius |
| Brain & Spinal Malformations | Q03.8 | Other congenital hydrocephalus |
| Brain & Spinal Malformations | Q04.0 | Congenital malformations of corpus callosum |
| Brain & Spinal Malformations | Q04.3 | Other reduction deformities of brain |
| Brain & Spinal Malformations | Q05.4 | Spina bifida, unspecified, with hydrocephalus |
| Brain & Spinal Malformations | Q05.6 | Thoracic spina bifida without hydrocephalus |
| Brain & Spinal Malformations | Q05.9 | Spina bifida, unspecified |
| Brain & Spinal Malformations | Q28.2 | Arteriovenous malformation of cerebral vessels |
| Brain & Spinal Malformations | Q67.4 | Other congenital deformities of skull |
| Brain & Spinal Malformations | Q87.0 | Malformation syndromes affecting facial appearance |
| Brain & Spinal Malformations | Q89.7 | Multiple congenital malformations |
| Neurological Sequelae of Infections | G93.4 | Encephalopathy, unspecified |
| Neurological Sequelae of Infections | G04.9 | Encephalitis/myelitis, unspecified |
| Neurological Sequelae of Infections | A85.1 | Adenoviral encephalitis |
| Neurological Sequelae of Infections | A86 | Unspecified viral encephalitis |
| Neurological Sequelae of Infections | G04.0 | Acute disseminated encephalitis |
| Neurological Sequelae of Infections | G05.1 | Encephalitis in diseases classified elsewhere |
| Neurological Sequelae of Infections | G61.0 | Guillain–Barré syndrome |
| Neurological Sequelae of Infections | I67.4 | Hypertensive encephalopathy |
| Progressive Metabolic Diseases | E72.5 | Disorders of glycine metabolism |
| Progressive Metabolic Diseases | E88.9 | Metabolic disorder, unspecified |
| Progressive Metabolic Diseases | E23.2 | Diabetes insipidus |
| Progressive Metabolic Diseases | E88.8 | Other specified metabolic disorders |
| Progressive Metabolic Diseases | E71.3 | Fatty-acid metabolism disorders |
| Progressive Metabolic Diseases | E83.3 | Phosphorus metabolism disorders |
| Progressive Metabolic Diseases | E83.5 | Calcium metabolism disorders |
| Progressive Metabolic Diseases | E10.1 | Type 1 diabetes with ketoacidosis |
| Progressive Metabolic Diseases | E10.8 | Type 1 diabetes with complications |
| Progressive Metabolic Diseases | E10.9 | Type 1 diabetes without complications |
| Progressive Metabolic Diseases | E11.8 | Type 2 diabetes with complications |
| Progressive Metabolic Diseases | E11.9 | Type 2 diabetes without complications |
| Progressive Metabolic Diseases | E13.1 | Other diabetes with ketoacidosis |
| Progressive Metabolic Diseases | E14.1 | Unspecified diabetes with ketoacidosis |
| Progressive Metabolic Diseases | E14.9 | Unspecified diabetes without complications |
| Progressive Metabolic Diseases | E72.2 | Urea cycle disorders |
| Progressive Metabolic Diseases | E74.2 | Galactose metabolism disorders |
| Progressive Metabolic Diseases | E78.8 | Other lipoprotein metabolism disorders |
| Progressive Metabolic Diseases | E83.1 | Iron metabolism disorders |
| Progressive Metabolic Diseases | E88.0 | Plasma-protein metabolism disorders |
| Neoplasms | C48.0 | Malignant neoplasm of retroperitoneum |
| Neoplasms | C49.4 | Malignant neoplasm of soft tissue of abdomen |
| Neoplasms | Q04.6 | Congenital cerebral cysts |
| Neoplasms | C74.9 | Malignant neoplasm of adrenal gland |
| Neoplasms | C22.2 | Hepatoblastoma |
| Neoplasms | C40.0 | Malignant neoplasm of scapula/long bones |
| Neoplasms | C41.4 | Malignant neoplasm of pelvic bones |
| Neoplasms | C64 | Malignant neoplasm of kidney |
| Neoplasms | C71.0 | Malignant neoplasm of cerebrum |
| Neoplasms | C71.7 | Malignant neoplasm of brain stem |
| Neoplasms | C71.9 | Malignant neoplasm of brain, unspecified |
| Neoplasms | C78.7 | Secondary malignant neoplasm of liver |
| Neoplasms | C79.0 | Secondary malignant neoplasm of kidney |
| Neoplasms | C79.5 | Secondary malignant neoplasm of bone/marrow |
| Neoplasms | C81.2 | Hodgkin disease, mixed cellularity |
| Neoplasms | C84.5 | Other mature T-cell lymphomas |
| Neoplasms | C91.0 | Acute lymphoblastic leukemia |
| Neoplasms | C92.0 | Acute myeloid leukemia |
| Neoplasms | C95.9 | Leukemia, unspecified |
| Neoplasms | D10.6 | Benign neoplasm of nasopharynx |
| Neoplasms | D17.7 | Benign lipomatous neoplasm, other |
| Neoplasms | D27 | Benign neoplasm of ovary |
| Neoplasms | D43.2 | Neoplasm of uncertain behavior of brain |
| Neoplasms | D48.7 | Neoplasm of uncertain behavior, other |
| Neoplasms | G93.0 | Cerebral cysts |
| Neoplasms | L98.0 | Pyogenic granuloma |
| Neoplasms | Z03.1 | Observation for suspected neoplasm |
| Cardiopathies | I42.8 | Other cardiomyopathies |
| Cardiopathies | Q24.8 | Other congenital heart malformations |
| Cardiopathies | I08.3 | Multiple valve disorders |
| Cardiopathies | I31.3 | Pericardial effusion |
| Cardiopathies | I40.0 | Infectious myocarditis |
| Cardiopathies | M30.3 | Kawasaki disease |
| Cardiopathies | Q21.3 | Tetralogy of Fallot |
| Cardiopathies | Q23.0 | Congenital aortic stenosis |
| Cardiopathies | Q23.2 | Congenital mitral stenosis |
| Cardiopathies | Q24.9 | Heart malformation, unspecified |
| Cardiopathies | Q25.1 | Coarctation of aorta |
| Congenital Immunodeficiencies | D58.9 | Hereditary hemolytic anemia, unspecified |
| Congenital Immunodeficiencies | G37.9 | Demyelinating disease of CNS |
| Congenital Immunodeficiencies | D59.1 | Autoimmune hemolytic anemias |
| Congenital Immunodeficiencies | D69.6 | Thrombocytopenia, unspecified |
| Congenital Immunodeficiencies | D80.0 | Hereditary hypogammaglobulinemia |
| Congenital Immunodeficiencies | D80.1 | Nonfamilial hypogammaglobulinemia |
| Congenital Immunodeficiencies | D82.0 | Wiskott-Aldrich syndrome |
| Congenital Immunodeficiencies | D84.9 | Immunodeficiency, unspecified |
| Congenital Immunodeficiencies | D89.2 | Hypergammaglobulinemia, unspecified |
| Congenital Immunodeficiencies | G35 | Multiple sclerosis |
| Congenital Immunodeficiencies | G37.3 | Acute transverse myelitis |
| Congenital Immunodeficiencies | G37.8 | Other demyelinating diseases |
| Congenital Immunodeficiencies | K50.0 | Crohn disease of small intestine |
| Congenital Immunodeficiencies | K50.1 | Crohn disease of large intestine |
| Congenital Immunodeficiencies | M32.1 | Systemic lupus erythematosus |
| Sickle Cell Disease | D57.1 | Sickle-cell anemia without crisis |
| Sickle Cell Disease | D57.0 | Sickle-cell anemia with crisis |
| Sickle Cell Disease | D57.2 | Double heterozygous sickling disorders |
| Sickle Cell Disease | D57.3 | Sickle-cell trait |
| Chromosomal Abnormalities | D82.1 | DiGeorge syndrome |
| Chromosomal Abnormalities | Q85.1 | Tuberous sclerosis |
| Chromosomal Abnormalities | Q90 | Down syndrome |
| Chromosomal Abnormalities | Q90.9 | Down syndrome, unspecified |
| Chromosomal Abnormalities | Q91.3 | Edwards syndrome, unspecified |
| Chromosomal Abnormalities | Q96.9 | Turner syndrome, unspecified |
| Chronic Kidney Failure | N11.1 | Chronic obstructive pyelonephritis |
| Chronic Kidney Failure | N12 | Tubulo-interstitial nephritis |
| Chronic Kidney Failure | N18.9 | Chronic kidney disease, unspecified |
| Digestive Tract Malformations | Q41.9 | Atresia/stenosis of small intestine |
| Digestive Tract Malformations | Q43.1 | Hirschsprung disease |
| Digestive Tract Malformations | Q43.3 | Malformations of intestinal fixation |
| Digestive Tract Malformations | Z90.4 | Acquired absence of digestive tract |
| Severe Osteogenesis Disorders | M86.9 | Osteomyelitis, unspecified |
| Severe Osteogenesis Disorders | M91.9 | Juvenile osteochondrosis |
| Severe Osteogenesis Disorders | Q75.0 | Craniosynostosis |
| Severe Osteogenesis Disorders | Q78.0 | Osteogenesis imperfecta |
| Severe Osteogenesis Disorders | Q78.8 | Other osteochondrodysplasias |
| Severe Osteogenesis Disorders | Q79.9 | Musculoskeletal malformation, unspecified |
| Transplant Complications | T86.8 | Complications of transplanted organs/tissues |
| HIV/AIDS | B20.3 | HIV disease with other viral infections |
| HIV/AIDS | B24 | HIV disease, unspecified |
| Epidermolysis / Dermatology | L51.1 | Stevens-Johnson syndrome |
| Epidermolysis / Dermatology | L51.8 | Other erythema multiforme |
| Cystic Fibrosis | E84.9 | Cystic fibrosis, unspecified |

**Legend:** ICD-10= International Statistical Classification of Diseases and Related Health Problems, 10th Revision
